# Ablation for paroxysmal atrial fibrillation—real-life results from a middle-volume electrophysiology laboratory

**DOI:** 10.1007/s10840-020-00937-1

**Published:** 2021-01-09

**Authors:** Piotr Kulakowski, Agnieszka Sikorska, Roman Piotrowski, Tomasz Kryński, Jakub Baran

**Affiliations:** Electrophysiology Laboratory, Department of Cardiology, Centre for Postgraduate Medical Education, Grochowski Hospital, Grenadierow 51/59, 04-073 Warsaw, Poland

**Keywords:** Atrial fibrillation, Ablation, Efficacy

## Abstract

**Introduction:**

A significant improvement in safety and efficacy of ablation for paroxysmal atrial fibrillation (PAF) has been reported by experienced centers over recent years; however, data from real-life surveys and smaller electrophysiology (EP) laboratories have been less optimistic.

**Aim:**

To asses efficacy of ablation for PAF in a middle-volume EP center over last years.

**Methods:**

Retrospective analysis of 1 year efficacy and safety of ablation for PAF in three cohorts of patients treated between 2011 and 2014 (period I), 2015–2017 (period II), and 2018–2019 (period III).

**Results:**

Of 234 patients (mean age 57 ± 9 years, 165 males), 81 (35%) were treated in period I, 84 (36%) in period II, and 69 (29%) in period III. The overall efficacy of ablation during all analyzed periods was 67%. The overall efficacy of ablation increased over time—from 56% in period I to 68% in period II and 81% in period III. Significant improvement was achieved using radiofrequency ablation (RF) (53% in period I vs 82% in period III, and 55% in period II vs 82% in period III, *p* = 0.003 and 0.0012, respectively) whereas positive trend in the improvement of cryoballoon efficacy was NS. The rate of peri-procedural complications was 9% and it did not change significantly over time.

**Conclusions:**

This real-life observational study from a medium volume EP center shows that progress in PAF ablation, especially RF, reported by highly-skilled centers, can be reproduced in real life by less experienced operators.

## Introduction

Ablation for paroxysmal atrial fibrillation (PAF) has been introduced into clinical practice over 20 years ago [1]. Pulmonary vein isolation (PVI) is a cornerstone of ablation in PAF [1]. A significant improvement in safety and efficacy of PVI has been documented by numerous scientific reports, generated by top-skilled and very experienced centers [2, 3]; however, data from real-life surveys have been less optimistic [4]. Operator’s growing experience, advances in technology, better understanding of AF mechanisms, and better patient’s selection have probably improved ablation success rate in everyday life in recent years; however, data are limited [3]. In addition, there was some criticism in the past that results of AF ablation were sub-optimal and not cost-effective [5]. In this context, data confirming significant improvement in AF ablation efficacy and safety in real world would be welcomed.

Additionally, in many countries, the vast majority of electrophysiology (EP) laboratories perform less than 50 AF ablations per year (in the USA even less than 20) [6]. A real-life success rates vary between 45 and 74% [4, 7] whereas major complication rate is increased in smaller centers, reaching 9% [6]. Therefore, it would be interesting to know whether novel technology and new ablation techniques improved ablation success rate in a real-life scenario in a typical middle-volume EP center.

## Aim

To asses efficacy of ablation for PAF in a middle-volume EP center over last years and to identify factors associated with success rate improvement.

## Methods

### Study group

Between June 2011 and October 2019, 775 ablation procedures for AF and 83 ablations for post-ablation left atrial tachycardia/flutter were performed in our center. Of these, 234 patients (mean age 57 ± 9 years, 165 males) were included in three prospective studies conducted in our laboratory [8, 9] in which the follow-up methodology was identical (NCT03811639, NCT03877913), enabling comparisons between groups of patients treated in various periods. All patients had to have PAF only and no previous AF ablation.

Three time-intervals were defined. The first time-period (period I) was set between June 2011 and February 2014. At that time, the first study [8] was conducted and the Navistar Thermocool SF (Biosence Webster, USA) ablation catheter with the CARTO 3 System (Biosence Webster, USA) was used for RF ablations whereas for cryoablation, the first generation of cryoballoon (CB) (Arctic Front 2AF281, Medtronic, USA) was used. The second time-period (period II) was set between October 2015 and December 2017 when the ABLANSAF study (NCT03811639) was conducted (9). At that time, the Thermocool SmartTouch catheter, CARTO 3 system with Visitag software (Biosence Webster, USA), was used for RF ablation and second generation of CB (Arctic Front 2AF283, Medtronic, USA) became available. The last time-period (period III) was set between January 2018 and October 2019 when the ABLANSAF study was ended and a new ongoing study AGNES (NCT03877913) was initiated. At that period, the Thermocool SmartTouch catheter, CARTO 3 system, ablation index, and CLOSE protocol were used and the use of second generation of CB was continued.

### Ablation methodology

During all analyzed periods, RF ablation was performed using double transseptal puncture for ablation catheter and circular mapping catheter. Changes in RF ablation methodology over time are described below. The CB ablation was performed in standard manner, using a long 8.5 F sheath and Brockenburgh needle for transseptal puncture, then exchanging it for a flexible 15F sheath (FlexCath Advance, Medtronic, USA) and inserting a balloon with diagnostic Achieve electrode (Medtronic, USA). One important difference between patients treated with RF vs CB was the presence of the common trunk of left PV. When this type of anatomy was detected on the pre-procedural cardiac computed tomography (CT) or intra-procedural intracardiac echocardiography (ICE), we used RF.

### Period I

RF ablation was performed with catheters without contact force sensing and the goal of ablation was to achieve PVI not paying too much attention to the quality and contiguity of RF applications which were manually marked on the CARTO map. We used power settings of 20–25 W at posterior wall and 30–35 W at anterior wall. All patients had pre-procedural computed tomography (CT) for left atrium (LA) imaging and subsequent merging into the CARTO system. At that time, ICE was used occasionally and only to guide transseptal puncture.

For CB, the first generation of balloons was used, the freezes had to be of 240–300 s duration, and two applications per one PV were performed. The Achieve catheter and pacing from PV were used to confirm PVI, and this approach did not change throughout all three analyzed periods. The nadir temperature achieved had to be less than − 36 °C and complete PV occlusion was attempted in each case.

### Period II

At this time-period, RF ablation changed significantly due to advent of contact force sensing ablation electrodes and further improvements in the CARTO system such as automatic tagging of ablation points (Visitag software). More attention was paid to the quality and contiguity of RF lesions; however, in case of durable PVI, leaving the gaps in the ablation line was still acceptable. The power settings remained unchanged. Also, catheter dragging instead of separate point-by-point technique was sometimes used by some operators. Pre-procedural LA imaging was mixed and consisted mainly of CT or rotational angiography which were subsequently merged with LA CARTO map. The ICE was used routinely for guiding transseptal puncture and for guiding ablation in some cases.

As far as CB was concerned, a new generation of balloons was introduced and more attention was paid to the quality of freezes, allowing for single application per vein and of shorter duration when PVI was achieved quickly (< 100 s) during first freeze.

### Period III

At that time, further improvements in RF methodology led to the implementation of the ablation index and CLOSE protocol into clinical practice. The RF lesions had to be of predefined quality (ablation index 350–400 at posterior wall and 450–500 at anterior wall) and closely spaced. The power settings remained unchanged; however, there was a clear end-point of each application—predefined ablation index. From the beginning of the use of ablation index, we decided to tag 2-mm dots instead of 3 mm advocated in the CLOSE protocol because we believed that such an approach further reduced the possibility of leaving gaps in the ablation line. We stopped to perform pre-procedural CT or rotational angiography and merging them with the CARTO map. Instead, we fully capitalized on our growing experience with ICE and used this modality to help in constructing CARTO map with ablation catheter and/or circular catheter. ICE was also used to guide ablation.

As far as CB is concerned, we started to use routinely time to PVI and thawing time as additional parameters to decide how long and how many freezes were needed to obtain PVI. Again, the use of ICE was expanded and it served for confirming complete PV occlusion in case of doubtful fluoroscopy images and to accurately position the Achieve catheter (not too deep in PV and not to proximal in LA).

### Other procedural details

All ablations were performed in patients in mild sedation with midazolam and fentanyl. The ACT was kept > 350 s during all analyzed periods. During period I, anticoagulants were stopped before the procedure and the patients were switched to low molecular weight heparin whereas in periods II and III, vitamin K antagonists were continued, preferably at the INR level of 2.0–3.0 whereas in those taking non-vitamin K anticoagulants, only the morning dose (directly before the procedure) was omitted and restarted 4 h after ablation. No protamine at the end of ablation was given. In period I, manual compression followed by bandage compression for 6 h was used whereas in periods II and III sites of punctures were sutured using a figure-of-eight suture and slight compression was instituted for approximately 6 h. The USG-guided vascular access has been used in our center since January 2017.

### Follow-up

All 234 patients were followed for 1 year in our outpatient clinic. They attended visits 3, 6, and 12 months after ablation during which history taking, physical examination and ECG were performed. All patients had 7-day Holter ECG recorded at these time-points. Ablation failure was defined as any recurrences of symptomatic AF or atrial tachycardia or any episode of AF > 30 s recorded during Holter ECG monitoring. If a patient was on antiarrhythmic drug prior to ablation, the drug was continued in the same dose for 3 months after the procedure and attending physicians were encouraged to stop antiarrhythmic medication at that point. Any increase in the dose of previously administered antiarrhythmic drug or introduction of new antiarrhythmic drug was regarded as ablation failure.

### Statistical analysis

The results are presented as mean ± SD or numbers and percentages. Quantitative data were compared using Student *t* test or ANOVA test whereas qualitative variables, using chi-square test with Yates’ correction or Fisher exact test. A *p* value < 0.05 was considered significant.

## Results

Of 234 patients, 81 (35%) were treated in period I, 84 (36%) in period II, and 69 (29%) in period III. Demographic and clinical characteristics of analyzed three groups of patients are compared in Table 1. There were no significant differences between the analyzed subgroups.

**Table 1:** Comparison of demographic and clinical parameters

Parameter	Period I *N* = 81	Period II *N* = 84	Period III *N* = 69	*p* value
Age (years)	57 ± 10	56 ± 10	59 ± 11	NS
Male gender (%)	58 (72%)	56 (67%)	51 (74%)	NS
BMI	29.8 ± 4	28.4 ± 4	28.1 ± 4	NS
CHA_2_DS_2_Vasc	1.14 ± 1.2	1.2 ± 1.2	1.6 ± 1.3	NS
HAS-BLED	0.17 ± 0.39	0.52 ± 0.63	0.54 ± 0.72	NS
LA size (mm)	38.7 ± 4.0	37.6 ± 4.5	38.0 ± 4.8	NS
LVEF (%)	66 ± 6	59.9 ± 5	59.3 ± 4	NS
Duration of AF history (years)	6.6 ± 5.2	5.6 ± 3.8	4.6 ± 3.0	NS

*BMI*, body mass index; *LA*, left atrial; *LVEF*, left ventricular ejection fraction

### Overall efficacy

**Fig. 1:**
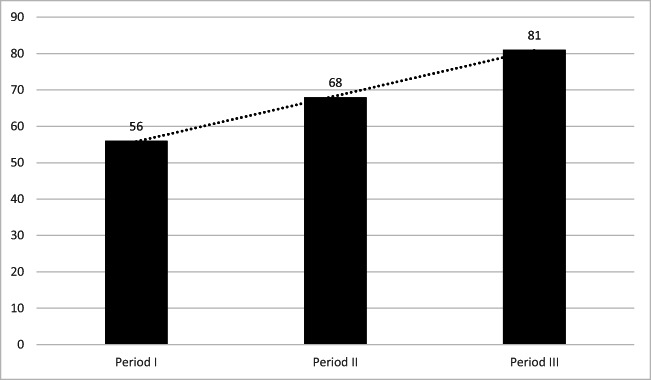
Overall efficacy (%) in analyzed time-periods. Period I vs period II and vs period III, *p*<0.05

The overall efficacy of ablation during all analyzed periods was 67% (158 patients). Of 123 patients treated with RF ablation, 79 (64%) were successfully treated compared with 79 (71%) of 111 patients who underwent CB (NS). The overall efficacy of ablation increased over time—from 56% in period I to 68% in period II and 81% in period III (Fig. 1). Of 158 effectively treated patients, 143 (91%) were off antiarrhythmic medication whereas the remaining 15 (9%) patients remained on unchanged doses of antiarrhythmic drugs.

Additional extra-PV ablation was performed in 4 patients from the RF arm (roof line in two, ablation of trigger in coronary sinus and fractionated potentials in one each). One patient treated with CB had RF touch-up application to finalize isolation of RIPV.

### RF ablation vs CB over time

**Fig. 2:**
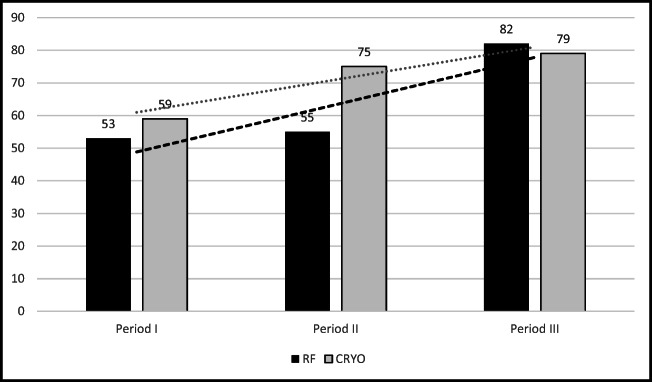
Comparison of RF vs CRYO efficacy (%) in analyzed time-periods. For RF: Period I vs period II and vs period III, *p*<0.01; for CB, NS

Comparison of success rate of RF ablation vs CB in analyzed time-periods is shown in Fig. 2. There was a constant increase in ablation efficacy over time using RF technique whereas improvement in CB was stagnant from period II onwards. Significant improvement was achieved using RF ablation (53% in period I vs 82% in period III, and 55% in period II vs 82% in period III, *p* = 0.003 and 0.012, respectively), whereas trend in improvement of CB efficacy was NS.

There were significant differences in the proportion of RF vs CB usage between period II vs periods I and III (49 vs 32 in period I, 29 vs 55 in period II, and 45 vs 24 in period III, respectively; *p* = 0.0008 for period I vs II, *p* = NS for period I vs III, and *p* = 0.0002 for period II vs III).

Changes in procedural details in RF group and CB group are shown in Tables 2, 3, and 4. There was a significant decrease in the fluoroscopy time, both using RF and CB whereas the total duration of procedure significantly shortened only in the CB group. The number of isolated PV increased over time using both techniques and reached all 4 PV in the last analyzed period. Analysis of RF details showed that total duration of RF time decreased in period II compared with period I and again increased during period III. The ablation count which depicts the number of RF applications showed similar changes with a marked increase during period III.

**Table 2:** Comparison of procedural details between three analyzed periods - RF

Parameter	Period I	Period II	Period III	P I vs II	I vs III	II vs III
Procedure duration (min)	247 ± 53	254 ± 64	237 ± 49	NS	NS	NS
Fluoroscopy time (min)	46.8 ± 16.4	22.1 ± 16.5	11.1 ± 6.0	< 0.05	< 0.05	< 0.05
RF details:						
RF duration (sec)	3735 ± 1316	2712 ± 1106	3381 ± 881	< 0.05	NS	< 0.05
Ablation count (number of RF applications)	103 ± 46	49 ± 19	121 ± 33	< 0.05	NS	< 0.05
Number of isolated PV	3.4 ± 1.1	3.9 ± 0.4	4	< 0.05	< 0.05	NS
ICE usage:						
For TP only	30 (61%)	26 (90%)	40 (89%)	0.007	0.002	NS
For TP and guiding procedure	0	13 (45%)	40 (89%)	0.0001	0.0001	0.0001

*RF*, radiofrequency; *PV*, pulmonary veins; *ICE*, intracardiac echocardiography; *TP*, transseptal puncture

**Table 3:** Comparison of procedural details between three analyzed periods - CB

Parameter	Period I	Period II	Period III	P I vs II	I vs III	II vs III
Procedure duration (min)	173 ± 43	144 ± 48	121 ± 18	< 0.05	< 0.05	NS
Fluoroscopy time (min)	44.8 ± 31.2	25.3 ± 14.4	15.9 ± 6.1	< 0.05	< 0.05	< 0.05
Number of isolated PV	3.6 ± 0.9	4	4	< 0.05	< 0.05	NS
Nadir temperature (°C):						
LSPV	− 48 ± 7	− 45 ± 6	− 44 ± 3	< 0.05	NS	NS
LIPV	− 48 ± 9	− 42 ± 5	− 40 ± 5	< 0.05	< 0.05	NS
RSPV	− 47 ± 8	− 48 ± 5	− 49 ± 5	NS	NS	NS
RIPV	− 42 ± 5	− 44 ± 5	− 43 ± 5	NS	NS	NS
Total number of freezes	9.3 ± 2.4	5.9 ± 1.8	5.5 ± 0.8	< 0.05	< 0.00001	NS
LSPV	2.7 ± 0.8	1.4 ± 0.6	1.4 ± 0.6	< 0.00001	< 0.00001	NS
LIPV	2.6 ± 1.0	1.7 ± 1.0	1.3 ± 0.7	< 0.00001	0.00002	NS
RIPV	2.0 ± 1.1	1.4 ± 0.7	1.4 ± 0.7	0.013	0.021	NS
RSPV	2.2 ± 0.9	1.5 ± 0.7	1.2 ± 0.4	0.0009	< 0.00001	0.02
Total freezing time sec)	2535 ± 862	1284 ± 329	1127 ± 191	< 0.05	< 0.05	NS
LSPV	719 ± 272	315 ± 137	262 ± 84	< 0.00001	< 0.00001	0.043
LIPV	705 ± 323	368 ± 174	280 ± 171	< 0.00001	< 0.00001	0.046
RIPV	538 ± 335	303 ± 136	288 ± 160	< 0.0007	< 0.0007	NS
RSPV	596 ± 301	304 ± 153	223 ± 90	< 0.00001	< 0.00001	0.005
ICE usage:						
For TP only	17 (53%)	21 (38%)	19 (79%)	NS	NS	0.0008
For TP and guiding procedure	0	5 (9%)	19 (79%)	NS	< 0.00001	< 0.0001

*LSPV*, left superior PV; *LIPV*, left inferior PV; *RIPV*, right inferior PV; *RSPV*, right superior PV; rest of abbreviations, as in Table 2

**Table 4:** Complications

Complications	Total *n*=21 (9%)	Period I *n*=8	Period II *n*=7	Period III *n*=6	RF/CB
Major:					
Cardiac tamponade	1	0	0	1	0/1
Significant peri-procedural pericardial effusion	2	2	0	0	2/0
Stroke	1	0	0	1	1/0
Transient ischemic attack	1	1	0	0	1/0
Right coronary artery air embolism following TP	1	1	0	0	0/1
Atrio-esophageal fistula	0	0	0	0	0
Death	0	0	0	0	0
Chronic pericarditis with large amount of pericardial fluid	1	0	0	1	1/0
Symptomatic PV stenosis	1	0	1	0	1/0
Persistent phrenic nerve paralysis	0	0	0	0	0
Pulmonary segmental atelectasis	1	1	0	0	0/1
Major subtotal	9	5	1	3	6/3
Local (before/after introduction of USG-guided VA)	12 (7/5)	3	6	3	8/4
Including:					
Arterio-venous fistula	6 (3/3)	1	3	2	4/2
Femoral artery pseudoaneurysm	2 (2/0)	1	1	0	2/0
Significant hematoma (requiring blood transfusion or prolonged hospitalization)	4 (2/2)	1	2	1	2/2

*VA*, vascular access; rest of abbreviations, Table 2

Using CB, isolation of all 4 PV was achieved using significantly lower number of freezes and shorter total freezing time per vein in periods III and II compared with period I. The value of nadir temperature slightly but significantly decreased in the left PV.

The usage of ICE for performing transseptal puncture and guiding ablation significantly increased over time both in the RF and CB arms.

### Complications

The rate of peri-procedural complications was 9%. There were no deaths (neither in-hospital or during follow-up). Also, no atrio-esophageal fistula or persistent phrenic nerve paralysis was noted. The most common were local complications with arterio-venous fistula being the most frequent. The rate of local complications did not significantly change after introduction of USG-guided vascular access (7/135 (5%) vs 5/99 (5%), NS) and tended to be higher in the RF than in the CB group (8/123 (7%) vs 4/111 (4%), NS). Two patients who suffered from TIA or stroke and a patient with cardiac tamponade (perforation of the right atrial appendage with a diagnostic electrode) recovered completely.

## Discussion

### Technological progress that increases ablation efficacy

During the analyzed period, a significant improvement in RF technology was introduced. They mainly included the use of contact force catheters and better control of the quality of RF lesions using the ablation index and CLOSE protocol [2]. These changes resulted in improvement in RF ablation efficacy, reported by experienced centers. We also observed a marked increase in efficacy of our procedures which shows that these new developments also work in real life in a middle-volume center. The only difference between our approach and that described in the original CLOSE protocol is that we used slightly lower values of ablation index; however, we used smaller dots representing RF applications on the CARTO map (2 mm instead of 3 mm). We believe that the use of smaller dots results in more dense ablation line around PV because more RF application have to be delivered without leaving gaps between RF applications. It is also encouraging that we did not see any excess in such complications as cardiac tamponade or PV stenosis when using higher density of RF applications. That may be influenced by fact that during all periods, non-surround flow catheter was used.

The increasing use of ICE during our procedures also might have increased our efficacy. This is the only currently available imaging technique which can be used in real time during ablation. Combination of ICE images and CARTO map created using ablation catheter or circular navigating catheter probably improves creation of real LA map and delineating PV ostia. It also helps to avoid RF energy delivery inside PV and is useful in early detection of such complications as steam pops or pericardial effusion [7].

The changes in the CB technique during the analyzed period were not so pronounced as in the RF technology. The biggest change was the introduction of new generation of cryoballoons which enabled more effective tissue cooling around PV ostia [10]. Another change was the assessment of lesion quality by introducing time to PVI and thawing time as important parameters for efficacy assessment. Also, duration of cryoapplications started to be more individualized, based on these parameters as well maximal temperature achieved and quality of PV occlusion [11].

### Learning curve and operator’s experience

Learning curve is important in any procedure. According to literature, learning curve in RF point-by-point technique is quite long and may require 100 procedures to perform as the first operator to obtain high skills [12]. This probably also influenced the results in our patients and resulted in higher efficacy in the last analyzed period. Between 2012 and 2019, five operators performed over 700 AF ablations in our institution and, in addition, over 300 procedures in five other institutions, where our team also works, which gives a total > 1000 AF ablations.

Learning curve in CB has been reported to be much shorter and only 20–30 procedures are enough to effectively and safely perform CB AF ablation procedures in patients with normal anatomy [13]. Some have even reported that the results may be similar in experienced and inexperienced centers [14]. However, there may be a significant difference in performance between an operator who is already experienced in RF AF ablation and an operator who just starts AF ablation procedures. Such anatomical issues as left PV common trunk, very small LA, additional PV, or very oval PV orifice may require more skilled operators [15]. Also, ICE images may help in proper balloon positioning in difficult cases [16].

### Changes in patients’ characteristics and procedural parameters over time

We did not observe any significant difference in patients’ demographic or clinical characteristics during analyzed periods. In spite of increasing knowledge that obesity and overweight significantly reduce efficacy of AF ablation [17], we did not manage to correct this parameter in our patients who had mean BMI between 28 and 31. This shows that more effort is required for risk factor modification, perhaps by especially dedicated teams [17].

### Changes in procedural parameters

Total duration of procedure reduced significantly in the CB group whereas it stayed constant in the RF group. The former result is expected and in line with data reported in literature [10, 13, 14] whereas no change in duration of RF ablation is unexpected and may be due to more detailed and meticulous delivery of lesions now than in the past in our laboratory. The overall time of approximately 4 h is long; however, it includes LA appendage assessment using ICE and approximately 20 min waiting time for PV reconnection, testing for PV conduction and searching for non-PV triggers.

The decrease in fluoroscopy time using both RF and CB is expected, in line with literature [3, 10, 13, 14], and reflects growing experience as well as advent of ICE, especially during RF ablation. After creating CARTO map, fluoroscopy is rarely used and almost all catheter movements and RF applications are guided by electro-anatomical map and ICE imaging. Also, an increase in the number of isolated PV which reached all 4 PV using either technique is in line with reported data [13, 14]. The reduction in fluoroscopy time in the CB arm between periods II and III was probably mainly due to the change of protocol from two freezes per vein to one freeze per vein. Further reduction in fluoroscopy time in the CB group between period II and III was mainly due to increasing overall experience, efforts to reduce fluoroscopic imaging time after contrast injection and, most importantly, expanding usage of ICE during the procedure (see Table 3) which is an excellent tool for confirming PV occlusion (color Doppler).

Differences in total RF duration and ablation count depict changes in the quality of RF lesions. We started with numerous lesions, however, with some being of poor quality (period I), then continued with lower number of lesions with better but not optimal quality (period II) and observed renewed increase in the RF application number and duration (period III) as a result of the CLOSE protocol introduction. In periods I and II, dragging technique was allowed which also might have influenced the lesion count—and resulted in less ablation counts with longer duration per application. In the last period (period III), we observed renewed increase in the RF application number and duration as a result of the CLOSE protocol introduction.

Lastly, small differences in nadir temperature achieved during CB in left PV may depict our growing awareness concerning possible PV stenosis and collateral damage when too low temperatures are achieved [18].

#### Complications

The frequency of complications was comparable to other real world reports [4, 6]. We believe that a low rate of cardiac tamponade in our study is in part associated with the ICE use. Two neurological complications occurred in patients with many risk factors (CHA_2_DS_2_Vasc = 5) or sub-optimal ACT during initial part of the procedure (maximal ACT of only 275 s was achieved). The rate of local complications was similar before and after introduction of USG-guided approach which shows that there is a room for improvement in our EP laboratory because data from literature suggest reduction of local complications when USG is used [19].

#### Limitations

This is a single-center experience with relatively low number of patients, however, with consistent follow-up scheme, completed by 100% of patients. We did not use implantable cardiac monitors to detect AF recurrences; thus, asymptomatic PAF may be under-detected. However, frequent clinical assessment and repeated 7-day Holter ECG recordings seem the most frequently used tools in other studies and in everyday clinical practice. A significant proportion of patients were on antiarrhythmic drugs during follow-up; however, the European registry also showed that a high proportion (46%) of patients remained on antiarrhythmic medication following AF ablation [4] so this depicts real-life situation. Follow-up duration was only 1 year—longer follow-up might have resulted in different results. There were differences in the proportion of RF vs CB usage between period II vs periods I and III which might have influenced the results. Extra-PV RF applications were performed in 4 patients from the RF group and one in the CB group. However, these numbers were really low (4 vs 1) and should have not influenced significantly the results.

## Conclusions

This real-life observational study from a medium volume EP center confirms results from high-volume very experienced centers on the progress in efficacy and safety of AF ablation, especially using RF energy, observed in recent years.
